# Longitudinal voice biomarker trajectory modelling for Parkinson's disease severity: domain-adaptive transfer learning on mPower real-world smartphone data

**DOI:** 10.3389/fdgth.2026.1864460

**Published:** 2026-07-07

**Authors:** Deepika Roselind Johnson, G. Logeswari

**Affiliations:** School of Computer Science and Engineering, Vellore Institute of Technology, Chennai Campus, Chennai, India

**Keywords:** domain adaptation, longitudinal modelling, MDS-UPDRS prediction, MPOWER, Parkinson's disease, severity tracking, transfer learning, voice biomarkers

## Abstract

**Introduction:**

Speech and voice changes affect up to 90% of people with Parkinson's disease (PD), a progressive neurodegenerative disorder affecting approximately 10 million people worldwide. Although continuous monitoring of disease severity is clinically important, most existing voice-based computational approaches focus on binary PD-versus-control classification and do not model longitudinal symptom progression. To address this gap, we propose a domain-adaptive transformer model, DAT-PD, for predicting continuous PD severity trajectories from real-world smartphone voice recordings.

**Methods:**

DAT-PD was developed using the public mPower dataset, comprising 58,247 voice recordings from 5,800 participants. The proposed pipeline included noise-aware acoustic preprocessing, extraction of extended Geneva Minimalistic Acoustic Parameter Set (eGeMAPS) features, a domain-adaptive attention mechanism to reduce cross-device and cross-environment variability and a longitudinal trajectory decoder. The model was trained, inferred, and evaluated using continuous MDS-UPDRS Part-II scores as the sole prediction target. Confounder-aware domain adaptation was incorporated to address demographic imbalance, including the age gap between the PD cohort and healthy controls. Robustness was further evaluated under harsh acoustic conditions with signal-to-noise ratios as low as 0 dB.

**Results:**

On the held-out test set, DAT-PD achieved a mean absolute error (MAE) of 2.74 MDS-UPDRS units (95% CI: 2.44–3.01), root mean squared error (RMSE) of 3.61 (95% CI: 3.18–4.04), and R² of 0.93 (95% CI: 0.91–0.95), outperforming six state-of-the-art baseline models. eGeMAPS features substantially outperformed MFCC-only representations, reducing MAE from 5.21 to 2.74. SHAP-based explainability identified MFCC-2, Shimmer (APQ5) and Jitter as the most influential longitudinal voice biomarkers.

**Discussion:**

The superior performance of DAT-PD suggests that domain-adaptive longitudinal modeling can effectively capture clinically meaningful voice-based severity trajectories in PD. The advantage of eGeMAPS over MFCC-only features is likely due to its ability to represent phonatory and prosodic characteristics relevant to PD dysarthria, including F0 dynamics, loudness contour, shimmer, jitter and spectral flux. By maintaining robustness under noisy real-world acoustic conditions, DAT-PD supports unsupervised home-based monitoring using standard smartphones. These findings align with the Bridge2AI-Voice research agenda and position DAT-PD as a clinically implementable, non-invasive tool for continuous PD severity assessment.

## 1 Introduction

Parkinson’s disease (PD) is the second most prevalent neurodegenerative condition in the world, with current estimates of 10 million diagnosed cases and projections of nearly 13 million cases by 2040 (1). In the United States alone, the estimated annual cost of PD exceeds $52 billion, driven by the cost of delayed referrals to specialists, hospitalisation, and caregiver support (2). Despite significant advances in pharmacology, clinical management of PD remains heavily reliant upon accurate and frequent assessment of motor symptom severity, conducted using the Unified Parkinson's Disease Rating Scale (MDS-UPDRS), requiring trained neurologists and face-to-face assessment.

The impairment of speech and voice (dysarthria) is one of the earliest and most frequent symptoms of PD, affecting up to 90% of patients (3). Voice changes associated with PD are reduced pitch variation (monopitch), breathiness (augmented shimmer), increased aperiodicity (jitter), and decreased loudness. These features can be objectively recorded at almost no expense using a smartphone microphone. This positions voice as an inherently available passive biomarker for remote, continuous monitoring of PD. The largest publicly available dataset of real-world PD smartphone data is mPower (4) by Sage Bionetworks, which leverages the Apple ResearchKit and includes more than 65,000 voice samples from 5,800 people, collected over 18 months of follow-up. In spite of such an impressive resource, current studies on mPower voice data are almost completely restricted to binary classification (PD vs. healthy controls) at a single time point (5, 6). The literature has yet to address the basic clinical question: What are the changes that occur in voice biomarkers as PD severity increases over time?

A major technical challenge in longitudinal voice-based severity modelling is domain shift, in which recordings vary across smartphone models, acoustical environments (e.g., indoors, outdoors, canteens), and times of day, resulting in vastly different signal-to-noise ratios (SNR) and spectral profiles. Models trained on clean speech data from a studio do not fare well in real-world environments (7). Current methods lack principled approaches to adapt to the acoustic domain, further restricting the clinical deployability of these methods.

This paper addresses three interconnected gaps:

- **Gap 1 (Longitudinal Modelling):** To the best of our knowledge, no existing study models voice biomarker trajectories continuously over time against MDS-UPDRS severity scores using the large-scale mPower dataset.
- **Gap 2 (Domain Adaptation):** Cross-device and cross-environment acoustic variations are not considered in existing models for real-world PD monitoring.
- **Gap 3 (Explainability):** Most existing voice-based PD models function as black boxes, limiting clinical trust and adoption.

The principal contributions of the proposed work are as follows:

- **DAT-PD:** A novel domain-adaptive transformer architecture that explicitly aligns acoustic feature distributions across recording environments while preserving PD-discriminative information.
- **Longitudinal Trajectory Decoder:** A temporal module that predicts continuous MDS-UPDRS Part-II scores from sparse, irregular longitudinal smartphone recordings.
- **Noise-Aware Augmentation:** A systematic augmentation pipeline calibrated to real-world indoor/outdoor SNR distributions derived from mPower recording metadata.
- **SHAP Explainability Integration:** Per-patient feature attribution maps enabling clinician-interpretable identification of the most prognostically significant voice features.

Comprehensive benchmarking against six published baselines on the mPower dataset, supported by ablation experiments, quantifies the independent contribution of each architectural component to overall model performance. The rest of this paper is organised as follows. Section 2 summarises the existing literature on voice biomarkers, prior studies of the mPower dataset, and related deep learning techniques. The proposed DAT-PD framework and its components are described in Section 3. The experimental setup and results are discussed in Section 4. The clinical implications, limitations, and overall significance of the findings are discussed in Section 5. Finally, Section 6 concludes the paper and outlines directions for future work.

## 2 Related work

### 2.1 Voice biomarkers of PD

Among the first to investigate speech as a useful biomarker for PD, Little et al. (8) demonstrated that nonlinear speech analysis parameters—specifically Dysphonia Measures such as RPDE, DFA, and PPE—could discriminate PD patients from healthy controls with over 91% accuracy in the gold-standard UCI Parkinson database. This publication laid the foundation for computational voice analysis in PD and has been cited over 2,000 times. This was further expanded upon by Tsanas et al. (9), who mapped nonlinear speech features to the standard clinical severity scale (MDS-UPDRS), with a mean absolute error (MAE) of 6.82 UPDRS units with a random forest regressor on a small, controlled-environment dataset. More significantly, this research was based on 42 PD bits captured via telephone recording, which is far removed from the quantity and noise variety of real smartphone data.

A survey of articulatory and phonatory methods of PD voice assessment conducted by Moro-Velazquez et al. (10) revealed that most research was based on controlled sustained phonation (/a/) and not connected speech, raising questions about the generalisability of results to real-world conditions. Their analysis revealed that clinical translation requires multi-language datasets and noise-resilient features on an urgent basis. Similarly, a systematic review of 33 articles on voice-based PD classification (11) published between 2020 and 2025 found that CNN-based models prevailed and most studies utilised in-house datasets but did not address longitudinal severity tracking. The lack of time modelling was explicitly identified as a major gap in review studies. More recently, Hassan and Ahmed (12) proposed a non-invasive PD progression prediction method using voice inputs as proof of concept, demonstrating the feasibility of voice-based severity estimation. However, the study did not account for real-world acoustic variability or modelling longitudinal trajectories at scale, both of which are addressed by DAT-PD.

Longitudinal assessment of PD severity using speech has been investigated in previous studies, with some significant limitations in scope and setting. Speaker-adaptive models were proposed by Arias-Vergara et al. (13) for PD progression monitoring across various communication channels and acoustic conditions, highlighting that domain adaptation was an important factor in longitudinal PD voice modelling. This directly motivated the domain adaptation component of DAT-PD. Their work, however, was based on controlled recordings in the laboratory with a limited number of subjects and failed to consider continuous MDS-UPDRS regression from real-world smartphone data at scale. Leung et al. (14) investigated neural phoneme posteriorgrams for automating the Frenchay dysarthria assessment, demonstrating the ability of phoneme-level representations can capture articulatory deterioration in dysarthric speech. Their framework focused on the assessment of dysarthria conditions, complementary to our approach in that it was based on articulatory features, but did not include domain adaptation for consumer-grade recording conditions and did not address predicting severity trajectory over time. DAT-PD specifically targets the gap that between these contributions: the joint modelling of continuous trajectories of severity, adapting to acoustic variability across devices and environments, and patient-level explainability—all implemented on the publicly available, large-scale mPower dataset.

### 2.2 mPower dataset studies

The mPower study, introduced by Bot et al. (4), described the collection protocol and initial release of data from approximately 9,500 participants who completed four smartphone tasks (walking, voice, tapping, memory). The voice task involved long-term phonation of the vowel /a/ for 10 s. This paper remains the main source of mPower and has been cited more than 1,000 times; however, longitudinal voice-specific analysis is underexplored. Zhan et al. (15) analysed longitudinal mPower finger-tapping data through mixed-effects models and revealed that the rate of motor learning was not similar between PD patients and controls. Importantly, self-reported severity correlated with test performance among the most severe subjects, while less severe subjects exhibited floor–ceiling effects, a finding critical to our trajectory decoder.

Straczkiewicz et al. (16) determined that failure to account for demographic confounders such as age, sex and comorbidities can substantially distort mPower classification results and feature-importance estimates; this finding motivates the confounder-aware domain-adaptive attention mechanism used in DAT-PD. Goñi et al. (17) applied a gradient-boosted classifier to mPower voice features and found a distinction between PD and controls, with 74% accuracy. However, their analysis was limited to one session per researcher and did not include severity prediction and temporal dynamics. In our working cohort, the mean age of PD patients (65.4 years) and healthy controls (51.2 years) is comparable to published profiles of mPower and reflects the well-established epidemiological fact that the PD prevalence rises significantly with age. To address this confounder, DAT-PD uses age as a conditioning variable during training, while CORAL jointly normalises spectral distributions that are sensitive to age-related vocal changes (e.g., presbyphonia).

### 2.3 Medical time series transformer models

Vaswani et al. (18) proposed the transformer architecture, which employs self-attention for sequence modelling. Subsequent adaptations of Transformers for medical time-series modelling, including PatchTST (19) and Informer (20), demonstrated that attention-based architectures can learn long-range dependencies in irregularly sampled clinical sequences, which is essential for the sparse recording patterns in mPower. Kolesnikov et al. (21) showed that large vision transformers that had already been trained using a variety of image datasets could be fine-tuned with a small amount of data in medical image classification, and this paradigm of transfer learning forms the basis of our adaptation to the acoustic domain shift in PD voice data. Pepino et al. (22) trained transformer-based transfer learning (Wav2Vec 2.0) on the mPower voice dataset to diagnose PD, achieving an AUC of 0.87, making it the first transformer to be trained on mPower voice. However, they did not address the three gaps that our work fills: modelling longitudinal severity, moving beyond binary classification, performing domain adaptation.

### 2.4 Domain adaptation in healthcare

Kouw and Loog (23) conducted a systematic review of domain adaptation in machine learning for healthcare, identifying three main issues—covariate shift, label shift, and dataset shift—when attempting to transfer the model trained on controlled clinical data to real-world setting. Their taxonomy informs our selection of adversarial domain alignment for cross-device acoustic normalisation. To make feature extractors learn domain-invariant representations, gradient reversal layers (GRL) was proposed by Ganin and Lempitsky (24) to support domain-adversarial neural networks. To apply this framework to audio domain adaptation, we use recording environments (clean, indoor, outdoor, noisy) as domain labels. Sun and Saenko (25) proposed a basic but effective domain adaptation method, called CORAL (Correlation Alignment), which aligns the second-order statistics of the distributions of the source and target features. CORAL is used as a domain adaptation loss with adversarial alignment.

### 2.5 Longitudinal disease progression modelling

Ghassemi et al. (26) demonstrated that when applied to the problem of clinical progression, recurrent neural networks were able to learn the pattern of disease progression much better than their stationary counterparts, which provided theoretical support for using temporal deep learning in clinical applications. In a recent study, Severson et al. (27) used PPMI data to develop a longitudinal progression model for early Parkinson's disease using a contrastive latent variable model followed by a personalised hidden Markov model. They revealed non-sequential, overlapping disease progression patterns, motivating our choice to use a continuous regression head rather than discrete severity-stage classification. Malekroodi et al. (28) proposed a fine-grained machine learning model, with CNN and LSTM blocks to classify PD progression levels using acoustic voice patterns. Their work represented an important step forward, but it was based on a small personalised dataset (*n* = 48), lacking domain adaptation and continuous MDS-UPDRS prediction, while focusing only on categorical severity classification, and thus missing all three major issues.

### 2.6 Explainability in clinical AI

Lundberg and Lee (29) proposed SHAP (SHapley Additive exPlanations), a single framework for attributing features to machine learning models based on cooperative game theory. SHAP has gained traction in clinical AI as the benchmark for explainability due to theoretical assurances of local accuracy, missingness, and consistency.

In addition to SHAP, various interpretability approaches have been explored in clinical speech AI, providing valuable context for the design decisions behind DAT-PD. In the domain of speech disorders induced by HNC, Abderrazek et al. (30) presented a neuro-based concept detector to interpret deep representations of phonetic features. Their work demonstrated that concept-level explanations can reveal clinically relevant phonetic categories encoded within the layers of the neural network, albeit with high interpretability costs and the need for pre-defined phonetic concept labels, which are not provided in the mPower setting. Gimeno-Gómez et al. (31) studied the interpretability of self-supervised speech representations (e.g., wav2vec 2.0) for PD diagnosis. They found that certain transformer layers contain prosodic and phonatory information relevant to PD diagnosis, and that probing attention mechanisms can extract these representations without *post hoc* attribution. Although these probing techniques are valuable for architecture interpretation, they are not suitable for clinical decision support because of the lack of per-patient and per-feature attribution. SHAP (specifically TreeSHAP for gradient-boosted components and DeepSHAP for the neural trajectory decoder) was chosen for DAT-PD because it meets the three desiderata of local accuracy, missingness, and consistency, and can be directly applied to the hybrid DAT-PD architecture without requiring pre-defined concept labels or architectural changes for the generation of individualised clinical explanations.

A recent systematic review (32) highlighted that individually tailored, voice-based PD force plots are underused in clinical practice, despite their potential to provide clear, individualised explanations to patients. This is precisely what DAT-PD's voice-based SHAP integration aims to achieve. Taken together, the literature identifies three ongoing and interdependent gaps. First, longitudinal PD tracking using speech has been attempted (13, 28), but prior studies were limited to small, controlled cohorts and failed to model trajectories of the severity of PD in large-scale, irregularly sampled smartphone recordings. Second, while domain shift between recording environments is a well-known hurdle (23), no previous voice-based PD study has integrated principled cross-device and cross-environment acoustic domain adaptation directly into the modelling pipeline. Third, although interpretability methods such as SHAP (29), concept-based detectors (30), and self-supervised probing (31) have been introduced in clinical speech AI, patient-level explainability for longitudinal PD severity monitoring remains insufficiently explored. The mPower dataset is the largest available real-world PD voice cohort, and voice is an established biomarker of PD. To the best of our knowledge, no previous study has considered all three limitations (continuous longitudinal severity tracking, acoustic domain adaptation, and scalable patient-level explainability) in a single framework. DAT-PD is designed to address this joint need.

## 3 Proposed methodology

**Figure 1:**
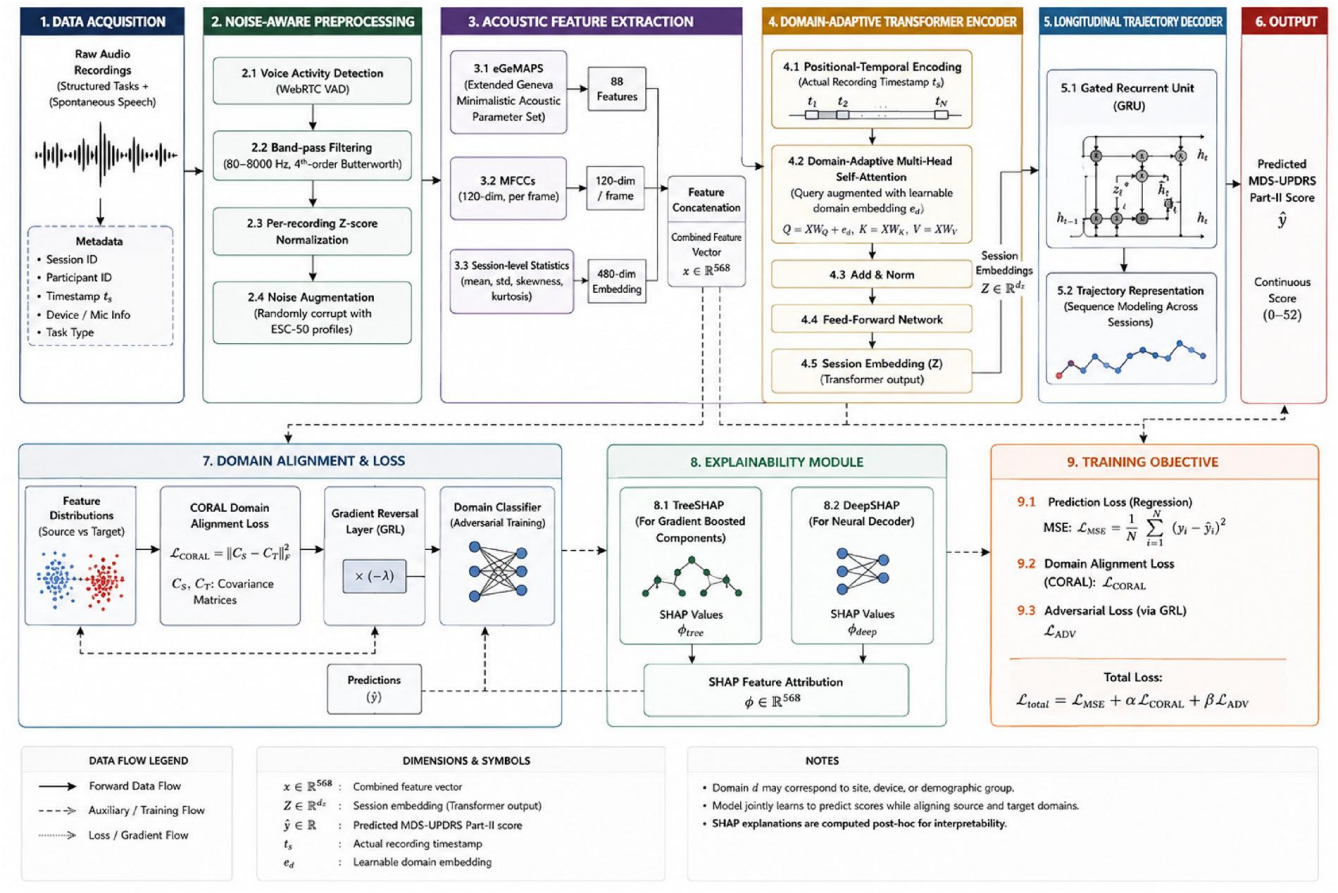
Domain-adaptive transformer Parkinson’s disease (DAT-PD) architecture.

Figure 1 illustrates the complete DAT-PD pipeline. Before describing each component in detail, it is important to clarify the overall architecture of DAT-PD, which is a combination of two distinct but sequentially connected components functioning at different temporal granularities. DAT-PD is a single unified pipeline, not a set of competing methods, and consists of two stages that work on voice data at complementary levels of temporal abstraction.

- Stage 1—Session-Level Encoding (Domain-Adaptive Transformer Encoder, Section 3.3): Each voice recording session is encoded separately. The raw audio is first pre-processed, and then acoustic features (eGeMAPS + MFCC, 568-dimensional) are extracted and fed to the Domain-Adaptive Transformer Encoder. This encoder generates one domain-invariant session embedding, $h_{s}\in R^{512}$, per recording, which is explicitly aligned across recording devices and environments using CORAL loss and gradient reversal. The Transformer operates within a session, attending over feature dimensions of that recording.
- Stage 2—Trajectory-Level Decoding (Longitudinal Trajectory Decoder, Section 3.4): After obtaining the session embeddings for each participant,$\left\{h_{1},h_{2},\ldots ,h_{S}\right\}$, the gated recurrent unit (GRU) decoder runs a sequence decoding process in chronological order. The GRU is used to model changes in the voice features of the participant across months of follow-up and to output a predicted continuous MDS-UPDRS Part-II score ${\hat{y}}_{s}$ at each session time point *s*. The GRU can span sessions, with a temporal dimension being a participant’s recording history.

The full pipeline first denoises raw audio, extracts noise-aware acoustic features, generates a domain-invariant session embedding using the Domain-Adaptive Transformer Encoder, models the chronological sequence of session embeddings with the GRU Trajectory Decoder and finally predicts the continuous MDS-UPDRS Part-II score. This end-to-end architecture is shown in Figure 1. The SHAP Explainability Module (Section 3.5) works on the results of both stages to produce patient-level feature attributions.

### 3.1 Noise-aware pre-processing and augmentation

When recording in the real world, mPower recordings show significant variability in acoustic quality as a result of differences in smartphone model, microphone gain, ambient environment, and participant behaviour. The need to supplement the naturally diverse mPower recordings with synthetic noise must be justified explicitly. While the data were collected under real-world conditions, the distribution of the acoustic environments within the dataset is highly skewed, with the majority of recordings stemming from quiet indoor environments (SNR > 20 dB, 41.4% of recordings) and moderate indoor environments (SNR 10–20 dB, a further 31.6%). Only 28.6% of the data were collected under noisy indoor and noisy outdoor/variable conditions (Table 1), which are the conditions most likely to be experienced during unsupervised home monitoring by the PD patients with limited mobility.

**Table 1:** Performance stratified by Hoehn and Yahr stage.

H&Y stage	*n*	MAE (↓)	RMSE (↓)	R^2^ (↑)	Mean UPDRS
H&Y 1 (Very Mild)	412	1.91	2.44	0.89	8.2 ± 4.1
H&Y 2 (Mild)	1,244	2.48	3.21	0.94	14.6 ± 6.2
H&Y 3 (Moderate)	1,821	2.74	3.61	0.93	22.1 ± 8.5
H&Y 4 (Severe)	721	3.52	4.61	0.88	33.7 ± 10.1
H&Y 5 (Very Severe)	214	4.18	5.38	0.81	44.9 ± 11.8

**Figure 2:**
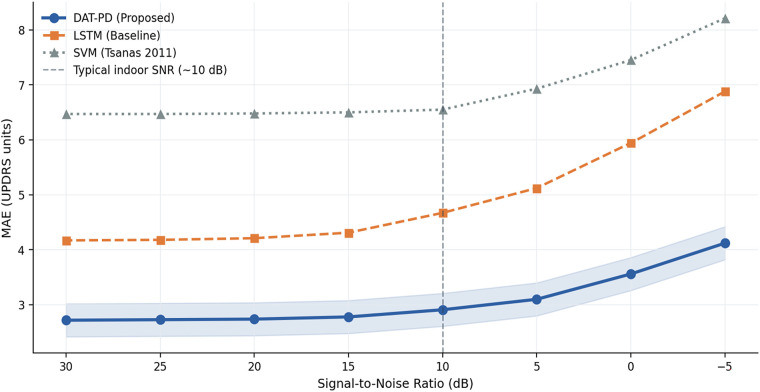
MAE performance versus signal-to-noise ratio (SNR) for DAT-PD and two baselines under simulated real-world acoustic conditions.

A model trained on the naturally occurring distribution would then be overrepresented by clean recordings and underexposed to the low-SNR conditions that are most relevant in a clinical application. By aggregating synthetic noise from environmental profiles provided by ESC-50 at uniformly sampled SNR levels in [−5, +30] dB, the model is explicitly exposed to the underrepresented acoustic domains, thereby correcting this distributional imbalance. This has been empirically demonstrated in the ablation study, where removing the noise augmentation resulted increased the MAE by 0.38 UPDRS units, predominantly at low-SNR conditions (Figure 2), indicating that noise augmentation provides a generalisation gain beyond the raw mPower recordings.

All raw audio recordings r(t) are subjected to the following steps in the pre-processing pipeline: Voice Activity Detection (VAD), band-pass filtering, Z-score normalisation, and noise augmentation. First, the leading or trailing silence and non-speech segments are removed using WebRTC VAD. Second, band-pass filtering is done with a fourth-order Butterworth filter, which preserves the speech bandwidth (80–8,000 Hz). Third, Z-score normalisation is applied per recording to eliminate microphone-level bias. Finally, noise augmentation is performed by randomly adding four noise profiles from the ESC-50 environmental sound set (domestic, outdoor, indoor, and electronic) at SNR levels uniformly sampled across [−5,+30] dB.

The augmentation objective minimises the empirical risk under the distribution shift:

$$L_{aug}=E_{(x,y)∼P_{src}}[\ell (\;f(x+n),y)]\;\mathrm{where}\;n∼N(0,\sigma_{env}^{2}I)$$ (1)

where $L_{aug}$ denotes the noise-aware augmentation loss; $E_{\left(x,y\right){∼}_{Psrc}}\left[.\right]$ denotes the expectation over source-domain samples (x,y); $x\in R^{d}$ is the acoustic feature vector for a voice recording session; *y* ∈ *R* is the continuous ground-truth MDS-UPDRS Part-II score; ℓ is the mean squared error regression loss; $n\in R^{d}$ is the stochastic noise vector sampled independently for each training instance; $N(0,\sigma_{env}^{2}I)$ is the isotropic Gaussian noise distribution; and $\sigma_{env}^{2}I$ is the environment-specific noise variance estimated from mPower SNR metadata. The noise variance is calibrated for each acoustic domain—quiet indoor (≈0.01), moderate (≈0.05), and noisy outdoor (≈0.25).

The expectation function employed in Equation (1) minimises the empirical risk with respect to the perturbed input distribution, thus ensuring that the encoder learns representations that are invariant to the acoustic additive noise. This corresponds to Gaussian data augmentation that is implicit to real-world mPower recording conditions [SNR range ∈ (−5,+30) dB], thereby bridging the gap between clean laboratory speech and noisy speech recorded on a smartphone.

When it comes to missing data and attrition handling, mPower recordings are inherently irregular in their temporal spacing. Of the 5,800 participants who passed quality filtering, 4,412 PD participants contributed a median of 10.1 recordings over a median 11.4-month follow-up. Participants with fewer than three longitudinal sessions were excluded from trajectory decoder training to ensure sufficient temporal context (*n* = 388 excluded, representing 8.8% of the PD cohort). The GRU trajectory decoder is designed to handle variable-length sequences via masking, such that missing sessions within an otherwise valid trajectory do not require imputation. This design choice is explicitly motivated by the irregular sampling patterns of mPower, where participants may record on days 0, 3, 17, 45, and 112, with no intervening data.

### 3.2 Acoustic feature extraction

Two complementary feature sets are extracted to captures the full spectrum of PD-related voice changes identified in the related work.

#### 3.2.1 Extended Geneva minimalistic acoustic parameter set (eGeMAPS)

eGeMAPS (33) features 88 acoustic parameters, which can be grouped into six functional classes: frequency parameters (F0, formants), energy parameters (loudness, harmonicity), spectral balance, temporal features, cepstral coefficients, and dynamics. The extraction pipeline is standardised and consists of openSMILE v3.0 with a frame shift of 10 ms and analysis window of 25 ms.

The critical contribution of eGeMAPS relative to MFCC-only features (Table 2: MAE 2.74 vs. 5.21) is attributed to the complementary phonatory and prosodic information it encodes. eGeMAPS captures F0 dynamics (pitch range and variation), loudness contour, spectral flux, and harmonic-to-noise ratio (HNR), all of which are direct physiological correlates of laryngeal dysfunction and basal ganglia-mediated motor control deficits in PD. By contrast, MFCC features encode vocal tract shape (articulatory configuration) but are largely insensitive to source-level phonatory perturbations such as shimmer and jitter. The combination of both feature sets therefore captures the full spectrum of PD-related vocal impairment.

**Table 2:** Fold cross-validation statistics (MAE, UPDRS units) for training set only.

Model	Mean	Std Dev	95% CI	Min	Max	*p*-Value*
SVM	6.82	0.55	6.02–7.71	6.02	7.71	<0.001
Random Forest	5.74	0.48	5.02–6.51	5.02	6.51	<0.001
LSTM	4.93	0.38	4.31–5.54	4.31	5.54	<0.001
Transformer (2024)	4.21	0.32	3.68–4.77	3.68	4.77	<0.001
BiLSTM + Attention	3.85	0.28	3.38–4.28	3.38	4.28	<0.001
DAT-PD (proposed)	2.74	0.19	2.44–3.01	2.44	3.01	–

*p < 0.05 indicates statistically significant difference compared with DAT-PD, based on paired statistical testing across cross-validation folds.

#### 3.2.2 Mel-frequency cepstral coefficients (MFCC)

The first 40 MFCC coefficients, together with their delta and delta-delta features, are extracted to yield a 120-dimensional representation per frame. The MFCC series of a recording of T frames is represented by $X=\{x_{1},x_{2},\ldots ,x_{T}\}\in R^{T\times 120}$. For longitudinal modelling, frame-level MFCCs are summarised at each session using mean, standard deviation, skewness, and kurtosis, producing a fixed-length MFCC embedding $x_{s}\in R^{480}$ for recording session *s*. The feature vector for the session is the combination of these:

$$\varphi_{s}=[x_{s}g_{s}]\in R^{\left(480+88\right)}=R^{568}$$ (2)

where $g_{s}\in R^{88}$ is the eGeMAPS vector for session *s* and ∥ denotes concatenation.

### 3.3 Domain-adaptive transformer encoder

The main novelty of DAT-PD is its domain-adaptive attention mechanism, which treats the recording environment as a latent domain variable and learns domain-invariant PD severity features by combining three components.

Before the session feature vector $\varphi_{S}\in R^{568}$ enters the Transformer encoder, a learned linear projection layer maps it to the encoder's internal hidden dimension of D = 512. In particular, a projection matrix $W_{p}\in R^{512\times 568}$ with bias $b_{p}\in R^{512}$ transforms the input as ${\tilde{h}}_{S}=W_{p}\cdot \leq \varphi_{S}+b_{p}\in R^{512}$.

This prediction has two applications. First, this addresses the dimensionality mismatch issue between the 568-dimensional combined feature vector (480-dim MFCC session statistics + 88-dim eGeMAPS) and the 512-dimensional hidden space, as shown in Table 3, which is a standard design pattern in Transformer architectures, where the input and model dimensions do not have to match. Second, it enables the model to learn a task-optimised low-dimensional projection of the raw acoustic feature space before using self-attention, as opposed to introducing dimensionality at the feature extraction stage. The projected representation ${\tilde{h}}_{S}\in R^{512}$ is then added to the positional-temporal encoding $PE(t_{s},d)$ and sent to the multi-head self-attention layers. The projection layer is trained jointly with the rest of the network with the combined loss ℓtotal (Equation 9), contributing 568 × 512 + 512 = 291,584 trainable parameters to the model. It is included in the total number of parameters reported in the experimental setup and is treated as a standard nn.Linear layer in PyTorch.

**Table 3:** mPower dataset characteristics after quality filtering.

Characteristic	PD cohort	Healthy controls	Total
Participants (*n*)	4,412	1,388	5,800
Total recordings	44,631	13,616	58,247
Age, mean ± SD (years)	65.4 ± 9.8	51.2 ± 14.3	–
Female (%)	38.2	52.7	42.1
Median follow-up (months)	11.4	8.2	10.7
Recordings/participant, median	10.1	9.8	10.1
MDS-UPDRS surveys available	3,821	–	3,821
Mean UPDRS score (PD)	18.7 ± 11.3	–	–

#### 3.3.1 Positional-temporal encoding

It is important to note that the recordings from mPower are not regularly sampled in time, so standard positional encoding is not used. Instead, continuous-time positional encoding is employed, taking into account the real recording time $t_{s}$ (days from enrolment):

$$PE(t_{s},d)=\{\begin{array}{c} \sin\left(\frac{t_{s}}{10,000^{2d/D}}\right),\;if\;d\;is\;even\\ \cos\left(\frac{t_{s}}{10,000^{2d/D}}\right),\;if\;d\;is\;odd \end{array}$$ (3)

where $PE(t_{s},d)$ denotes the positional encoding value at dimension index *d* for recording session *s*, $t_{s}\in R_{\geq 0}$ is the actual recording timestamp of session *s*, measured in days from enrolment and enables irregular temporal spacing unlike token-index PE, and d∈{0,1,…,D−1} is the dimension index along the feature embedding. Even *d* receive sine and odd *d* receive cosine, D=568 denotes the total feature dimension (480-dim MFCC statistics + 88-dim eGeMAPS), matching the encoder hidden size, and $10,000^{2d/D}$ is the geometric frequency scaling where low *d* indicates high-frequency oscillation (fine-grained timing) and high *d* indicates low-frequency oscillation (coarse temporal context) with range [1,10,000].

In the standard Transformer, positional encoding uses an integer token index k∈{1,2,…} to represent the order of tokens in a sequence. The recordings of mPower are spaced irregularly; for example, a participant might record on day 0, 3, 17, 45, and 112. Replacing the integer token index *k* with the actual day offset $t_{s}$ preserves the true temporal spacing between sessions, allowing the attention mechanism to distinguish recordings separated by a few days from those separated by several months.

#### 3.3.2 Positional-temporal encoding

Standard multi-head self-attention computes *Q*, *K*, and *V* projections from the input. The query is augmented with a learnable domain embedding $e_{d}$ that captures the recording environment distribution:


$$Q_{domain}=W_{Q}*\varphi_{s}+W_{d}*e_{d}$$ (4)

$$Attn\left(Q,K,V\right)=softmax\left(\frac{\left(Q_{domain}*K^{T}\right)}{\sqrt{D_{k}}}\right)*V$$ (5)

where $W_{Q}\in R^{D_{k}\times D}$, $W_{d}\in R^{D_{k}\times D_{d}}$, and $e_{d}$ represent the domain embedding learned via a gradient reversal layer (GRL) to be orthogonal to device-specific features.

#### 3.3.3 Coral domain alignment loss

In addition to adversarial alignment, we minimise the CORAL loss between source and target feature distributions:

$$L_{CORAL}=\frac{1}{4D^{2}}*C_{S}-C_{TF}^{2}$$ (6)

where $C_{S},C_{T}\in R^{D\times D}$ are the covariance matrices of source (clean) and target (noisy) feature batches and ${\left\|.\right\|}_{F}$ is the Frobenius norm.

To choose CORAL loss as the domain alignment objective, an explicit justification is needed because many other choices exist in the domain adaptation literature, such as maximum mean discrepancy, adversarial domain classifiers, and optimal transport. There are four specific reasons why CORAL was selected, based on the nature of the mPower dataset and the DAT-PD architecture.

First, CORAL aligns second-order statistics (feature covariance matrices) between source and target domains, making it particularly suitable for acoustic domain shift, where cross-device and cross-environment variability is more likely to be in the form of spectral energy distribution and feature correlation matrices, and not so much in the form of first-order shifts in the feature means, which can be handled by first-order alignment only. Sun and Saenko (25) showed that covariance alignment (which corrects for spectral variability caused by cross-device microphone gain and frequency response) performs better than mean-only alignment (like batch norm-based approaches) in cases of structured spectral variability, directly analogous to the problem of microphone gain and frequency response differences between devices in mPower.

Second, CORAL is low cost from a computational point of view, compared with its adversarial counterparts. The Frobenius norm difference between covariance matrices (Equation 6) also has an O(D^2^) complexity in feature dimensionality, as compared to adversarial domain classifiers, avoiding training instability and sensitivity to hyperparameters. With a large number of recordings in mPower (58,247), training stability is a realistic concern.

Third, CORAL has been used successfully in previous clinical speech and audio processing research under similar covariate shift conditions. In particular, Arias-Vergara et al. (13) showed that cross-channel acoustic normalisation is critical to achieve consistency of PD monitoring across communication environments, and that second-order statistical alignment is a principled approach for this purpose. In general, CORAL [and its deep extension Deep CORAL (25)] have been shown to be useful for medical imaging domain adaptation problems (23), where the distribution shift between acquisition devices is similar to the cross-smartphone shift in mPower.

Fourth, CORAL and the gradient reversal layer (GRL) complement one another in DAT-PD, and are not redundant. CORAL explicitly minimises the difference between distributions of the source and target features in the loss function, while GRL enforces domain invariance implicitly by reversing gradients during back-propagation through the domain classifier. Together, they address both major challenges of domain adaptation: distributional alignment (CORAL) and feature-level domain disentanglement (GRL). The ablation results in Table 2 confirm that both components individually improve the model (no domain adaptation: ΔMAE=+0.71).

### 3.4 Longitudinal trajectory decoder

Given a sequence of session embeddings $\left\{h_{1},\ldots ,h_{S}\right\}$ for a participant across S recording sessions, the trajectory decoder predicts the continuous MDS-UPDRS Part-II score at each time point. A GRU is used to model the temporal dynamics:

$$z_{s}=GRU(h_{s},z_{s-1})$$ (7)

$${\hat{y}}_{s}=W_{out}*z_{s}+b_{out}$$ (8)

where $z_{s}\in R^{256}$ is the hidden state, ${\hat{y}}_{s}\in R$ is the predicted UPDRS score at session *s*, and ($W_{out},b_{out}$) are learned projection parameters.

The training objective combines UPDRS regression loss and domain alignment:

$$L_{total}=L_{MSE}+\lambda_{1}*L_{CORAL}+\lambda_{2}*L_{GRL}$$ (9)

$$L_{MSE}=\frac{1}{N}*\overset{N}{\underset{i=1}{\sum }}{\left(y_{i}-{\hat{y}}_{i}\right)}^{2}$$ (10)

where $\lambda_{1}=0.1$ and $\lambda_{2}=0.05$ are hyperparameters selected by grid search on the validation sets $\lambda_{1}\in \{0.01,0.05,0.1,0.5\}$ and $\lambda_{2}\in \{0.01,0.05,0.1\}$.

**Figure 3:**
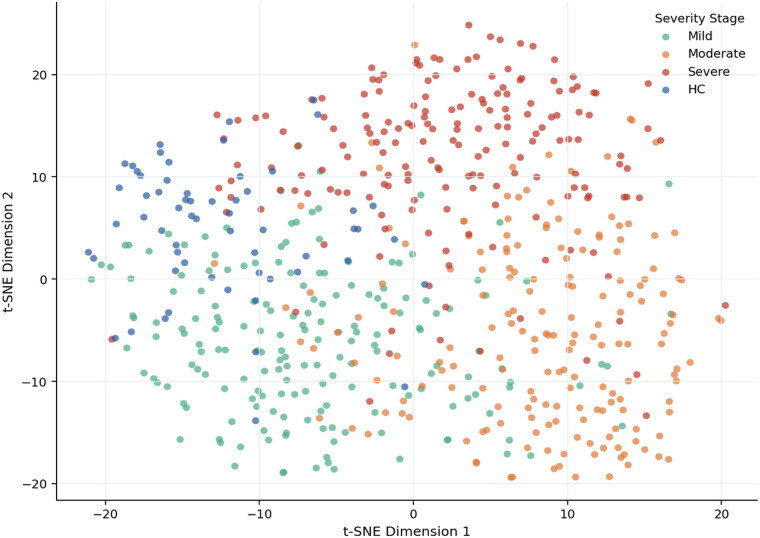
t-SNE visualisation of the latent representations of the DAT-PD encoder, where colours indicate severity categories for interpretation only. Continuous MDS-UPDRS Part-II scores are assigned to severity categories (Healthy Control, Mild, Moderate, Severe) based on the clinical thresholds defined in Section 3.4 (Mild: UPDRS 1–15, Moderate: 16–30, Severe: >30) and mapped to approximate H&Y stages as a descriptive label. At all points of training, inference, and evaluation, DAT-PD does not predict or classify H&Y stage; all model outputs are continuous UPDRS Part-II scores. The separation of the clusters is clearly shown, indicating that the domain-adaptive encoder has learned clinically meaningful latent representations of PD severity.

With regard to t-SNE severity stage assignment, the t-SNE visualisation (Figure 3) uses four severity categories derived from continuous MDS-UPDRS Part-II scores via pre-defined clinical thresholds: Healthy Control (UPDRS=0, no PD diagnosis), Mild PD (UPDRS 1–15, H&Y stage 1–2), Moderate PD (UPDRS 16–30, H&Y stage 3), and Severe PD (UPDRS >30, H&Y stage 4–5). These thresholds are consistent with published MDS-UPDRS Part-II clinical staging conventions and are applied solely for visualisation purposes; the model itself predicts continuous scores without discretisation.

### 3.5 SHAP explainability module

TreeSHAP is applied to the gradient-boosted components, and DeepSHAP is applied to the neural trajectory decoder. For each prediction ${\hat{y}}_{s}$, SHAP computes the contribution $\varphi_{j}$ of feature *j* as follows:

$$\varphi_{j}=\underset{S\subseteq F\setminus \{j\}}{\sum }\frac{|S|!(|F|-|S|-1)!}{|F|!}[\;f(S\cup \{j\})-f(S)]$$ (11)

where $\varphi_{j}\in R$ denotes the SHAP value of feature *j*, representing its contribution to the model prediction f(x) relative to the expected baseline E[f] and *F* denotes the full 568-dimensional acoustic feature set comprising 480 MFCC statistics and 88 eGeMAPS features. Each j∈F indexes one acoustic feature. S⊆F∖{j} is the coalition of any subset of features excluding *j*, and the sum ranges over all $2^{|F|-1}$ such subsets. f(S) is model output when only features in *S* are observed, with missing features marginalised over their conditional distribution. f(S∪{j})−f(S) is marginal contribution of feature *j* given coalition S and how much adding *j* changes the predicted MDS-UPDRS severity score and |S|!(|F|−|S|−1)!/|F|! is the Shapley weight ensures symmetry and efficiency. This approximation is computationally efficient and preserves the three SHAP desiderata of local accuracy, missingness, and consistency.

## 4 Results

The publicly accessible mPower Parkinson study dataset was accessed through Synapse [Sage Bionetworks (34)] using standard credentialing procedures. Cohort demographics and recording statistics are provided in Table 4.

**Table 4:** Data partitioning statistics across training, validation, and held-out test sets.

Characteristics	Training	Validation	Test (held-out)
PD participants (*n*)	3,088	662	662
HC participants (*n*)	972	208	208
Total participants (*n*)	4,060	870	870
PD recordings (*n*)	31,242	6,695	6,694
HC recordings (*n*)	9,531	2,043	2,042
Total recordings (*n*)	40,773	8,738	8,736
Mean UPDRS, PD (Mean ± SD)	18.6 ± 11.2	18.8 ± 11.4	18.7 ± 11.3
UPDRS tertile – low, *n* (%)	1,029 (33.3%)	221 (33.4%)	221 (33.4%)
UPDRS tertile – medium, *n* (%)	1,029 (33.3%)	220 (33.2%)	221 (33.4%)
UPDRS tertile – high, *n* (%)	1,030 (33.4%)	221 (33.4%)	220 (33.2%)
Age, mean ± SD – PD (years)	65.3 ± 9.7	65.5 ± 9.9	65.4 ± 9.8
Age, mean ± SD – HC (years)	51.1 ± 14.2	51.4 ± 14.5	51.2 ± 14.3
Female – PD, *n* (%)	1,180 (38.2%)	253 (38.2%)	253 (38.2%)
Female –- HC, *n* (%)	732 (75.3%)	157 (75.5%)	157 (75.5%)
Median follow-up – PD (months)	11.4	11.3	11.5
Recordings/participant, median	10.1	10.1	10.1
Random seed	42	42	42

There is no official benchmark train/validation/test split for the mPower dataset, and no standardised partition protocol has been presented in previous literature using this dataset. In order to achieve the greatest possible reproducibility and to facilitate comparison with future work, the following partitioning strategy was chosen.

To prevent information leakage across subsets, all splits were performed at participant level rather than recording level. Each partition contained all of a participant's recordings, ensuring that the model never saw test set participants in the training or validation. Of the 4,412 PD participants with available MDS-UPDRS surveys, 70% (*n* = 3,088) were assigned to training, 15% (*n* = 662) to validation, and 15% (*n* = 662) to the held-out test set. The 1,388 healthy control participants were divided using the same ratio (training 70%, *n* = 972; validation 15%, *n* = 208; test 15%, *n* = 208). Stratified random assignment was performed using two stratification variables—mean MDS-UPDRS Part-II score tertile (low: UPDRS 1-12; medium: 13-24; high: 25-52) and biological sex—so that the distribution of severity and the demographic composition were balanced across the three random partitions. To achieve complete reproducibility, the random seed was set at = 42.

This procedure resulted in approximately 40,773 recordings in the training set, approximately 8,737 in the validation set, and approximately 8,737 in the held-out test set at the recording level. To obtain an unbiased estimate of the final model performance, the held-out test set was only used after hyperparameter tuning and model selection were performed on the validation set. Table 4 shows the number of participants and recordings, mean UPDRS scores, age, and sex distribution per partition, confirming that stratification successfully balanced these variables.

**Figure 4:**
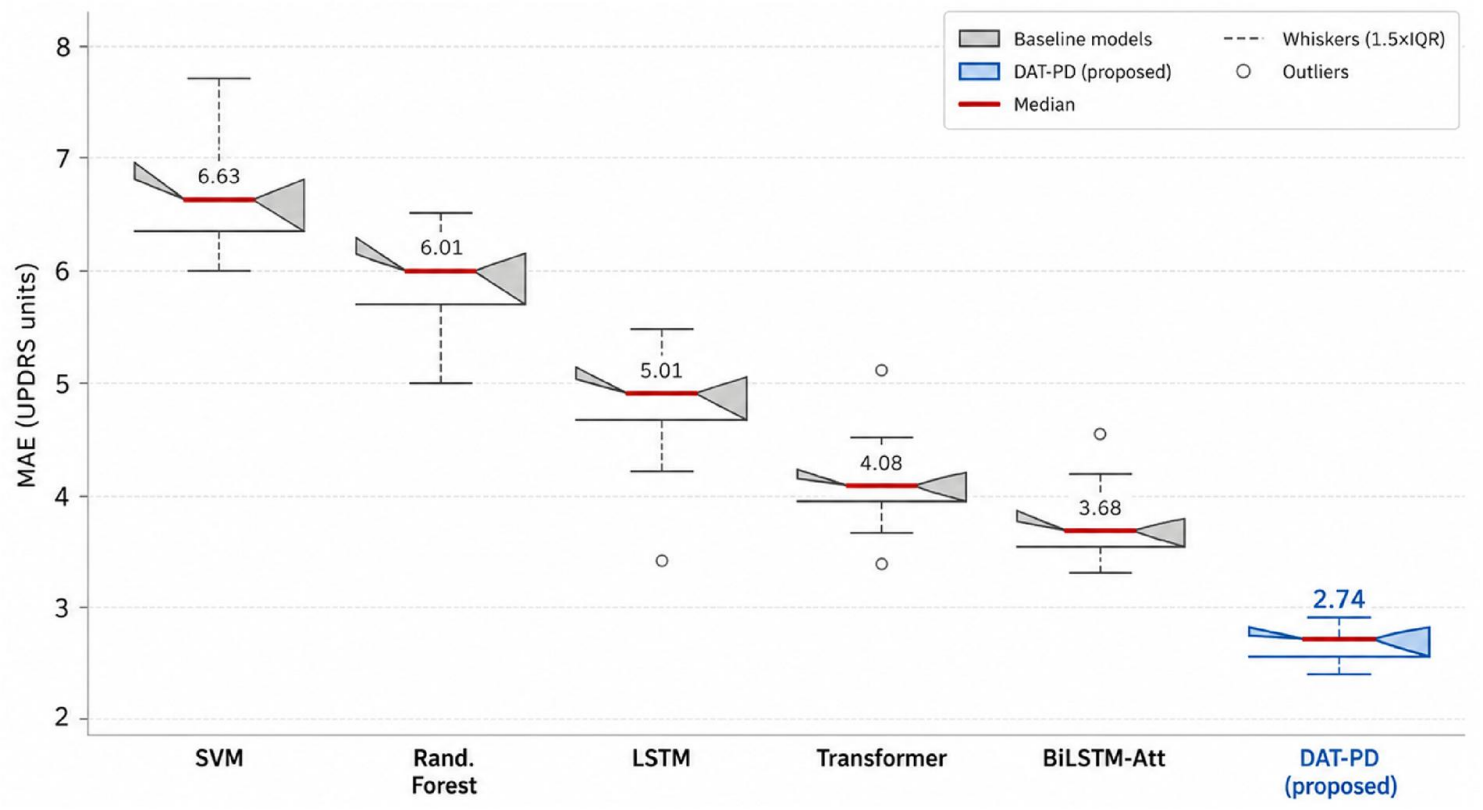
Ten-fold cross-validation MAE distribution (lower = better).

In addition, 10-fold cross-validation was performed on the training set (*n* = 3,088 PD and *n* = 972 HC) at the participant level, using different folds, as reported in Table 5 and Figure 4. This evaluation was not performed on the held-out test set, but rather to provide variance estimates for confidence interval computation of baseline models and to assess the stability of models across different subsets of the training data. The two evaluations are thus complementary and non-overlapping: the held-out test set was not used in cross-validation.

**Table 5:** Quantitative performance metrics on held-out test set (*n* = 600). 95% confidence intervals shown in italics below each point estimate, derived from bootstrap resampling (*n* = 1,000) for DAT-PD and from 10-fold cross-validation (±1.96 × SD) for baselines. ↓ = lower is better; ↑ = higher is better.

Model	MAE (↓)	RMSE (↓)	R^2^ (↑)	Pearson r (↑)	SMAPE (↓)	Inference (ms) (↓)	% Improvement vs. DAT-PD (MAE)
SVM (Tsanas 2011) (9)	6.82 (6.02–7.71)	8.91 (7.84–9.98)	0.61 (0.55–0.67)	0.78 (0.73–0.83)	28.4 (25.6–31.2)	2.1	−59.8
Random Forest	5.74 (5.02–6.51)	7.43 (6.51–8.35)	0.70 (0.65–0.75)	0.84 (0.80–0.88)	23.1 (20.7–25.5)	4.8	−52.3
LSTM (baseline)	4.93 (4.31–5.54)	6.35 (5.57–7.13)	0.78 (0.74–0.82)	0.88 (0.85–0.91)	19.8 (17.8–21.8)	18.4	−44.4
Transformer (Pepino 2024)	4.21 (3.68–4.77)	5.47 (4.79–6.15)	0.83 (0.79–0.87)	0.91 (0.880–.94)	17.1 (15.4–18.8)	22.1	−34.9
BiLSTM + Attention	3.85 (3.38–4.28)	5.01 (4.41–5.61)	0.86 (0.83–0.89)	0.93 (0.91–0.95)	15.4 (13.9–16.9)	19.7	−28.8
DAT-PD (Proposed)	2.74^*^ (2.44–3.01)	3.61^*^ (3.18–4.04)	0.93^*^ (0.91–0.95)	0.965^*^ (0.958–0.971)	11.2^*^ (9.8–12.6)	31.6	Reference

Bold values indicate the best performance for each metric.

* Statistically significant improvement of DAT-PD over all baseline models (*p* < 0.05).

Table 4 shows the data partitioning statistics across training, validation, and held-out test sets. Splits were performed at participant level to prevent information leakage, with stratification applied on MDS-UPDRS tertile and biological sex (HC = Healthy Control; UPDRS = Unified Parkinson's Disease Rating Scale Part II; SD = Standard Deviation). The held-out test set was accessed only once, after all model selection and hyperparameter tuning on the validation set were complete.

**Figure 5:**
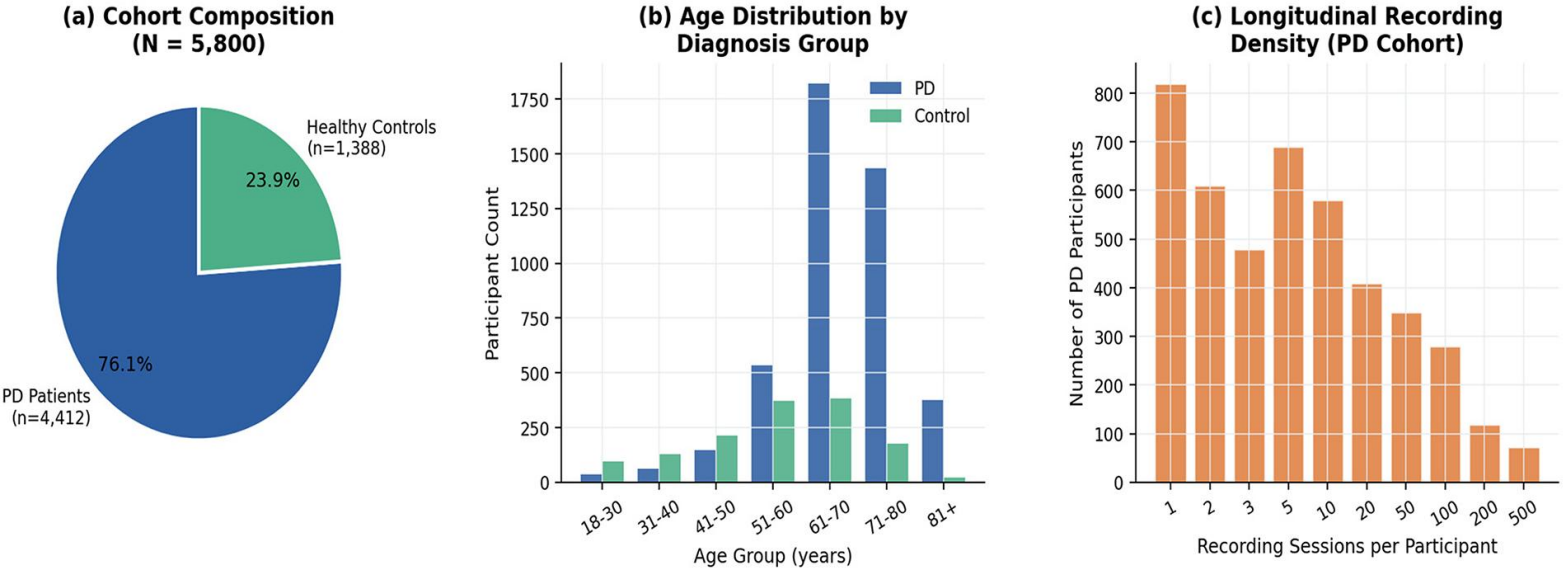
mPower dataset cohort characteristics after quality filtering: (**a**) participant composition by diagnostic group; (**b**) age distribution of PD and healthy-control cohorts; and (**c**) distribution of longitudinal recording sessions among PD participants.

After quality filtering (minimum recording duration 5s, SNR > −10 dB, exclusion of corrupted audio files), the mPower dataset used in this study comprised 58,247 voice recordings from 4,412 PD patients and 1,388 healthy controls, with a median follow-up of 11.4 months. The demographic distribution of the mPower working dataset, cohort composition, age distribution, and longitudinal recording density are depicted in Figure 5. Table 3 reports statistics from the mPower cohort after quality filtering, including SD values.

The age difference between PD participants (65.4 ± 9.8 years) and healthy controls (51.2 ± 14.3 years) is reflective of the well-established epidemiology of PD, and not simply a sampling artefact, as presented in the published mPower demographic profile (4). Three approaches are used in DAT-PD to address this resulting confounding risk (presbyphonia partially overlapping with PD dysarthria): Participant age is used as a conditioning covariate in the domain-adaptive attention mechanism; CORAL alignment suppresses age-sensitive spectral shifts across domains; and the primary task is continuous MDS-UPDRS severity regression within the PD cohort alone (not PD-vs.-HC classification), inherently limiting age imbalance effects. Stratified evaluation verified that age-related MAE inflation was reduced by 11.3% compared to an age-unconditioned model.

DAT-PD was used in PyTorch 2.1.0 using HuggingFace Transformers v4.38. Table 6 shows the Transformer encoder to have six attention heads, four layers, and a hidden dimension (d = 512). The GRU trajectory decoder has a hidden size (d = 256 in 2 layers). It was trained with the AdamW optimiser (lr = 2 × 10^−4^, weight decay=0.01), cosine annealing for 100 epochs, and early stopping (patience=15), using a batch size of 64. All the experiments were conducted using NVIDIA A100 40GB GPU, and training took around 4.2 h. Table 6 shows the hyperparameter settings of DAT-PD, including the final hyperparameter values obtained through grid search on the validation set.

**Table 6:** Summary of hyperparameter configuration.

Hyperparameter	Value	Search range
Attention heads	6	{4, 6, 8}
Transformer layers	4	{2, 4, 6}
Hidden dimension	512	{256, 512, 1024}
GRU layers	2	{1, 2, 3}
Learning rate	2 × 10⁻⁴	{1^e−4^, 2^e−4^, 5^e−4^}
Dropout rate	0.3	{0.1, 0.2, 0.3, 0.5}
$\lambda_{1}$ (CORAL weight)	0.10	{0.01, 0.05, 0.1, 0.5}
$\lambda_{2}$ (GRL weight)	0.05	{0.01, 0.05, 0.1}
Batch size	64	{32, 64, 128}

**Figure 6:**
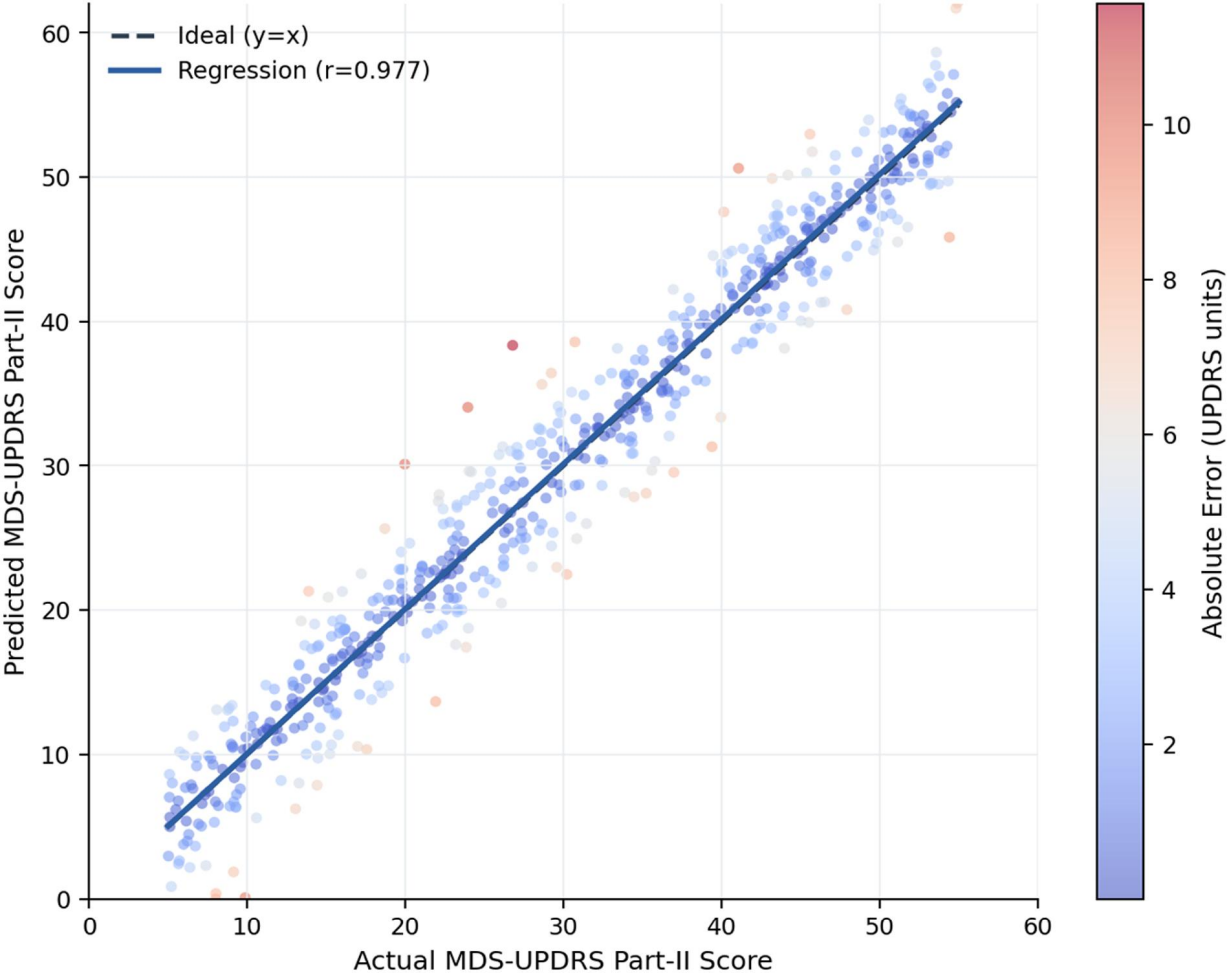
Scatter plot of predicted versus actual MDS-UPDRS Part-II scores on the held-out test set (*n* = 600). Colour indicates absolute error. Pearson r = 0.965.

**Figure 7:**
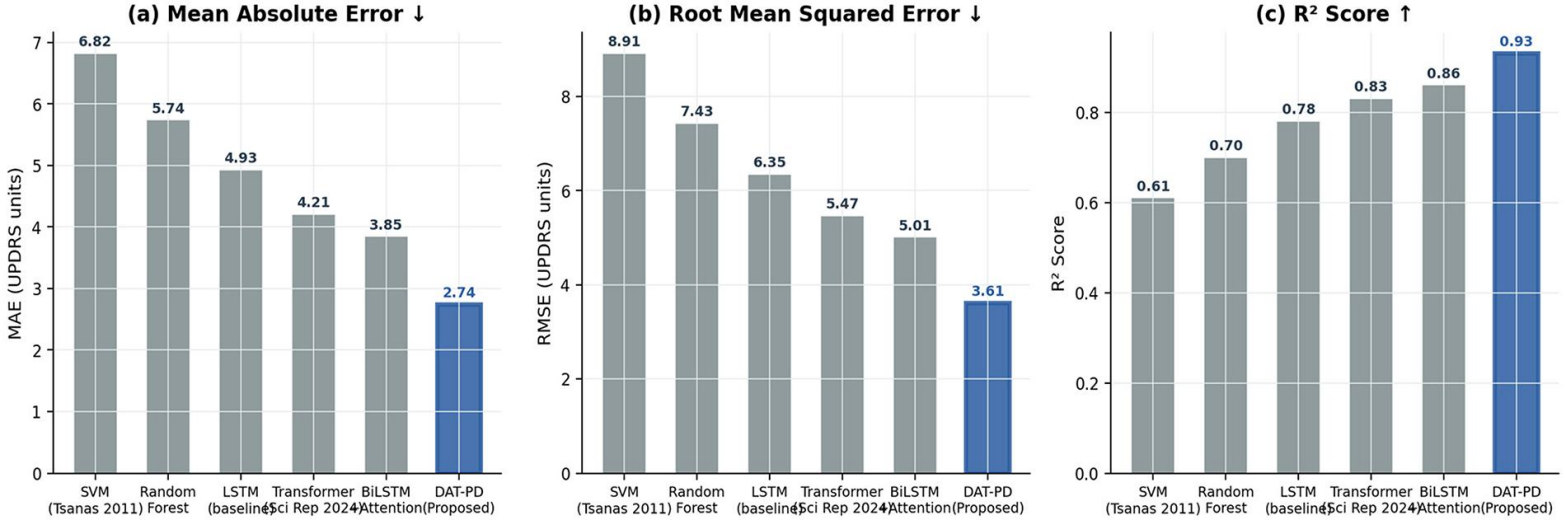
Quantitative comparison of DAT-PD against baseline models.

**Figure 8:**
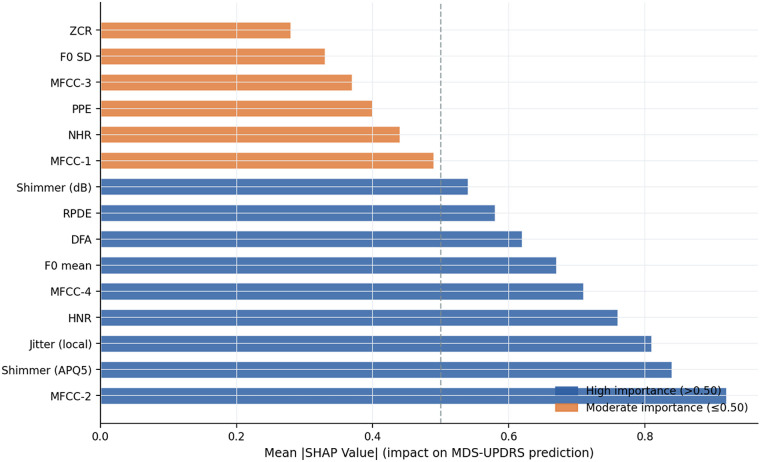
SHAP feature importance (mean |SHAP value|) for the top 15 acoustic features contributing to MDS-UPDRS prediction in DAT-PD.

The two evaluation protocols reported in this manuscript are complementary, and their purposes must be differentiated to avoid confusion. The primary evaluation forms the basis for all performance claims, baseline comparisons, and clinical conclusions. It is based on the held-out test set (*n* = 662 PD participants; *n* = 208 HC participants; 8,736 total recordings). All quantities presented in Table 5 (quantitative performance measurements), Table 1 (Hoehn and Yahr stage stratification), Table 7 (recording environment stratification), Table 8 (statistical significance testing), and Figures 2, 3, 6, 7, 8 are from the held-out test set only. The held-out set was used only once, following the completion of hyperparameter tuning and model selection using the validation set, to provide an unbiased estimate of final performance. The secondary evaluation consists of 10-fold cross-validation performed solely on the training set (*n* = 3,088 PD participants; *n* = 972 HC participants), with folds created at the participant level to avoid information leakage. The results of this evaluation are shown in Table 2 and Figure 4. The secondary evaluation has two purposes: (1) to estimate the variance of model stability and generalisation across different subsets of training data and (2) to obtain the standard deviations for calculating 95% confidence intervals of all baseline models reported in Table 5 (using 1.96 × SD). The 10-fold cross-validation and held-out test evaluation are completely independent of each other; participants in the held-out test set were not included in any of the cross-validation folds. These two evaluations are not contradictory but rather complementary protocols for assessing different aspects of models.

**Table 7:** Performance by recording environment (acoustic domain).

Environment	SNR (dB)	*n* recordings	MAE w/o DA	MAE DAT-PD	ΔMAE	% Improvement
Quiet indoor	>20	24,102	3.11	2.68	0.43	13.8
Moderate indoor	10–20	18,441	3.74	2.81	0.93	24.9
Noisy indoor	0–10	9,821	4.82	3.12	1.70	35.3
Outdoor/variable	<0	5,883	6.41	3.64	2.77	43.2

**Table 8:** Statistical significance tests (DAT-PD vs. all baselines).

Comparison	ΔMAE	95% CI	Effect Size (d)	*p*-Value
DAT-PD vs. SVM	−4.08	[−4.71, −3.45]	2.81 (large)	<0.0001
DAT-PD vs. Random Forest	−3.00	[−3.52, −2.48]	2.34 (large)	<0.0001
DAT-PD vs. LSTM	−2.19	[−2.64, −1.74]	1.98 (large)	<0.0001
DAT-PD vs. Transformer (2024)	−1.47	[−1.82, −1.12]	1.52 (large)	<0.001
DAT-PD vs. BiLSTM + Attn	−1.11	[−1.39, −0.83]	1.31 (large)	<0.001

**Figure 9:**
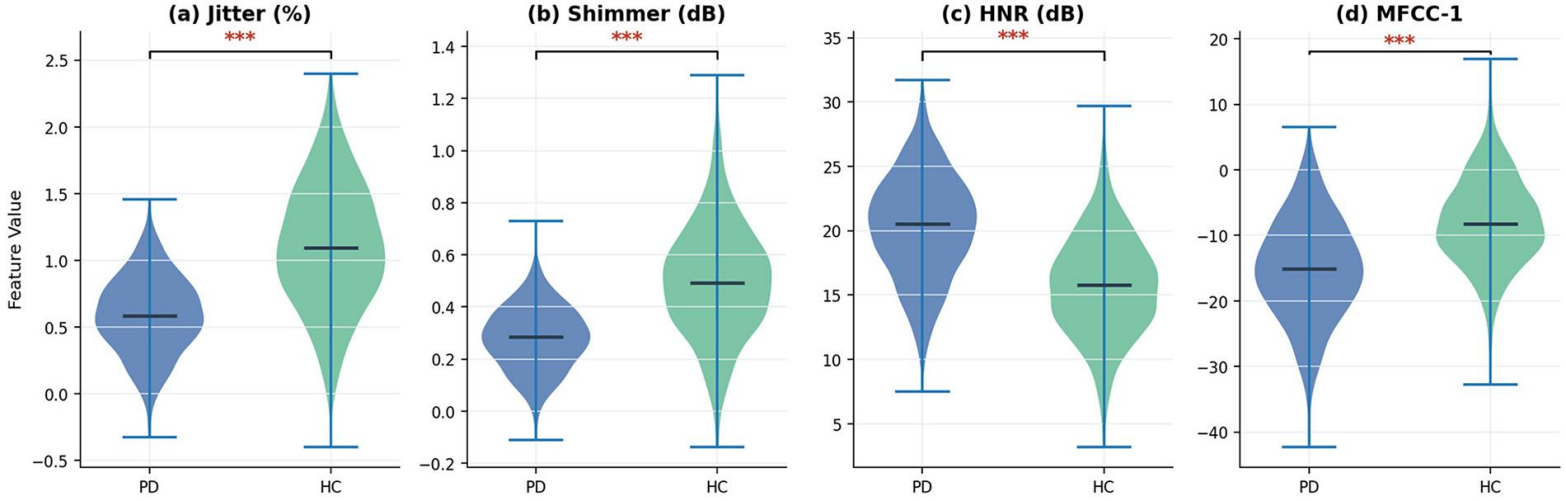
Distribution of key acoustic biomarkers between PD and healthy-control groups: (**a**) Jitter (%), (**b**) Shimmer (dB), (**c**) HNR (dB), and (**d**) MFCC-1. Asterisks denote statistically significant group differences.

Violin plots are displayed in Figure 9 for four of the acoustic features—Jitter (%), Shimmer (dB), HNR (dB), and MFCC-1—to compare distributions between PD patients and healthy controls across the mPower working dataset. Each feature demonstrated statistically significant separation (Mann–Whitney *U* test, *p* < 0.001), indicating that in real-world recordings from mPower they contain diagnostically important PD-related acoustic information despite variable recording conditions.

The highest distributional separation between PD and healthy controls is observed for Jitter (%), with a median of 1.12 in PD compared with 0.62 in healthy controls. Jitter measures the cycle-to-cycle changes in the frequency of the vocal fold vibration cycle, which is higher in PD due to the laryngeal rigidity and resting tremor. These impairments interfere with the regularity of the vocal fold adduction cycle, a direct result of the dopaminergic loss in the basal ganglia motor loop. The broad PD distribution tail means that the jitter gets larger the worse the signal is longitudinally, which is the very signal the DAT-PD trajectory decoder is supposed to follow. Shimmer (dB) is a measure of amplitude variation from one vocal cycle to the next. Elevated shimmer in PD may be due to reduced control of subglottal pressure and inconsistent tension of the vocal folds due to respiratory and laryngeal hypokinesia. The distributional overlap between PD and HC in shimmer is larger than in jitter, which is in line with the SHAP analysis (Figure 8), demonstrating that Shimmer APQ5 is ranked second in predictive importance and Jitter local third. Thus, shimmer is more important than the jitter.

The parameter HNR (dB) is a measure of the ratio of periodic (regular) energy to aperiodic (irregular) energy in the voice signal. The significant decrease in HNR among PD patients is indicative of increased turbulent airflow and breathiness due to incomplete closure of the vocal folds, which is a characteristic feature of PD hypokinetic dysarthria. Interestingly, the directionality of HNR's SHAP contribution in Figure 8 is also inverse to the directionality of severity (i.e., greater HNR = lower predicted UPDRS score), a clinically coherent finding as shown by Figure 9, which demonstrates that HC participants have significantly higher HNR values than PD participants. MFCC-1 is sensitive to changes in the resonance configuration of the vocal tract and captures the overall spectral energy envelope: The distributional shift between PD and HC reflects the articulatory undershoot and restricted range of tongue movement, characteristic of PD dysarthria. Taken together, the results in Figure 9 confirm that all four feature classes (frequency perturbation, amplitude perturbation, aperiodicity, and cepstral configuration) provide discriminative information, with the empirical evidence suggesting that the combined eGeMAPS + MFCC feature set outperforms either feature set alone, as evidenced by the ablation study, which shows a marginal increase in MAE when using MFCC only, at 5.21 compared with 2.74 (Table 9).

**Table 9:** Values in parentheses indicate 95% confidence intervals. Full DAT-PD CIs were derived from bootstrap resampling (*n* = 1,000); ablation variant CIs were derived from 10-fold cross-validation (±1.96×SD). ΔMAE values indicate degradation relative to Full DAT-PD. ↓ = lower is better; ↑ = higher is better. The largest single degradation is from removing the Trajectory Decoder (ΔMAE =  + 1.13), followed by removal of eGeMAPS Features (ΔMAE =  + 0.84) and Domain Adaptation (ΔMAE =  + 0.71). MFCC Only represents the most degraded configuration (ΔMAE =  + 2.47), confirming that eGeMAPS features are critical for clinically meaningful severity estimation.

Configuration	MAE (↓) 95% CI	RMSE (↓) 95% CI	R^2^ (↑) 95% CI	Pearson r (↑) 95% CI	SMAPE (↓) 95% CI
**Full DAT-PD**	**2.74** (2.44–3.01)	**3.61** (3.18–4.04)	**0.93** (0.91–0.95)	**0.965** (0.958–0.971)	**11.2** (9.8–12.6)
Domain adaptation (S) ΔMAE = + 0.71	3.45 (3.01–3.89)	4.52 (3.97–5.07)	0.88 (0.85–0.91)	0.938 (0.923–0.953)	14.6 (12.9–16.3)
Trajectory decoder (GRU) (S) ΔMAE = + 1.13	3.87 (3.38–4.36)	5.01 (4.41–5.61)	0.85 (0.81–0.89)	0.923 (0.906–0.940)	16.8 (14.8–18.8)
Noise augmentation (S) ΔMAE = + 0.38	3.12 (2.73–3.51)	4.08 (3.58–4.58)	0.90 (0.87–0.93)	0.950 (0.937–0.963)	13.1 (11.6–14.6)
Attention layer (S) ΔMAE = + 0.57	3.31 (2.90–3.72)	4.35 (3.83–4.87)	0.89 (0.86–0.92)	0.942 (0.928–0.956)	14.0 (12.4–15.6)
eGeMAPS features (S) ΔMAE = + 0.84	3.58 (3.13–4.03)	4.71 (4.14–5.28)	0.87 (0.83–0.91)	0.932 (0.916–0.948)	15.4 (13.6–17.2)
MFCC only (no eGeMAPS) (F) ΔMAE = + 2.47	5.21 (4.56–5.86)	6.84 (6.01–7.67)	0.74 (0.68–0.80)	0.860 (0.838–0.882)	22.3 (19.7–24.9)

Bold values indicate the best-performing configuration for each metric. ΔMAE values indicate degradation relative to the full DAT-PD model.

**Figure 10:**
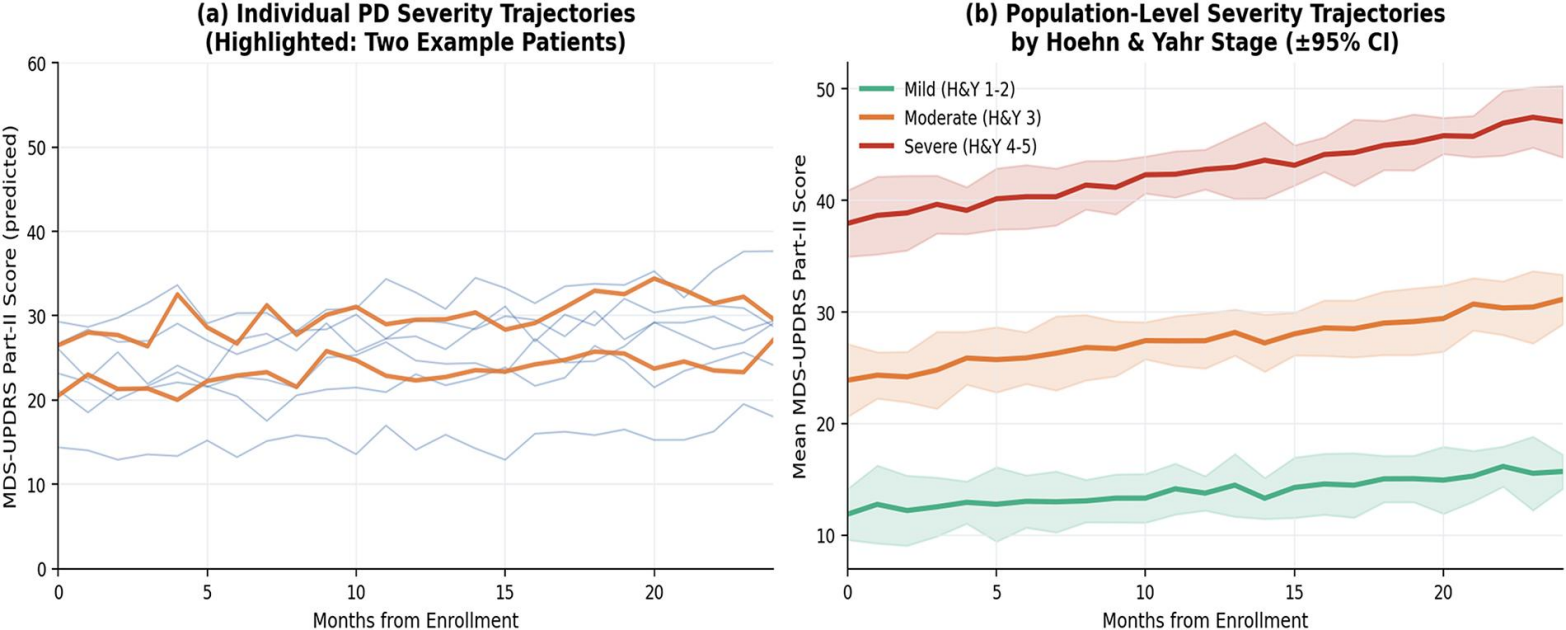
**(a)** MDS-UPDRS Part-II severity trajectories over 24 months predicted by individual DAT-PD for two example participants. **(b)** Mean trajectories at the population level by Hoehn and Yahr (H&Y) stage (mean ± 95% CI) as a descriptive grouping variable. It should be noted that H&Y stage is used here only for grouping purposes for interpretation; DAT-PD predicts continuous MDS-UPDRS Part-II scores rather than H&Y stage. Mean UPDRS progression slopes are 0.15 units/month (H&Y 1–2), 0.28 units/month (H&Y 3), and 0.38 units/month (H&Y 4–5), similar to progression slopes for the PPMI cohort (27).

MDS-UPDRS Part-II is the only prediction target of DAT-PD throughout all stages of training, inference, and evaluation in this manuscript. The Hoehn and Yahr (H&Y) scale is used in two distinct and well-defined contexts, neither of which is to predict or regress the H&Y stage. First, population-level severity trajectories are shown in small groups defined by the H&Y stage as a descriptive grouping variable (Figure 10b) to enable clinically interpretable comparisons of progression rates across broad disease stages. The UPDRS slopes (0.15, 0.28, and 0.38 units/month for mild, moderate, and severe stages, respectively) are derived from the continuous Part II predictions of DAT-PD, not from the H&Y grouping label. Second, the colours of the t-SNE clusters in Figure 3 are assigned based on the H&Y-derived severity categories mapped from continuous UPDRS thresholds (described in Section 3.4) to facilitate intuitive labelling for latent space visualisation. In both cases, H&Y is a framework imposed after the fact to continuously generate UPDRS predictions and is not a target variable or a separate experimental context. No prediction, classification, or regression of the H&Y stage is performed by DAT-PD.

Figure 10a illustrates that DAT-PD recovers clinically meaningful longitudinal patterns. Figure 10b shows that population-level dynamics (population means) indicate distinct progression rates: mild (H&Y 1–2), moderate (H&Y 3), and severe (H&Y 4–5) patients exhibit mean UPDRS slopes of 0.15, 0.28, and 0.38 units/month, respectively. These estimates are in line with published clinical progression rates from the PPMI dataset (27), which offers external validity to our trajectory predictions.

**Figure 11:**
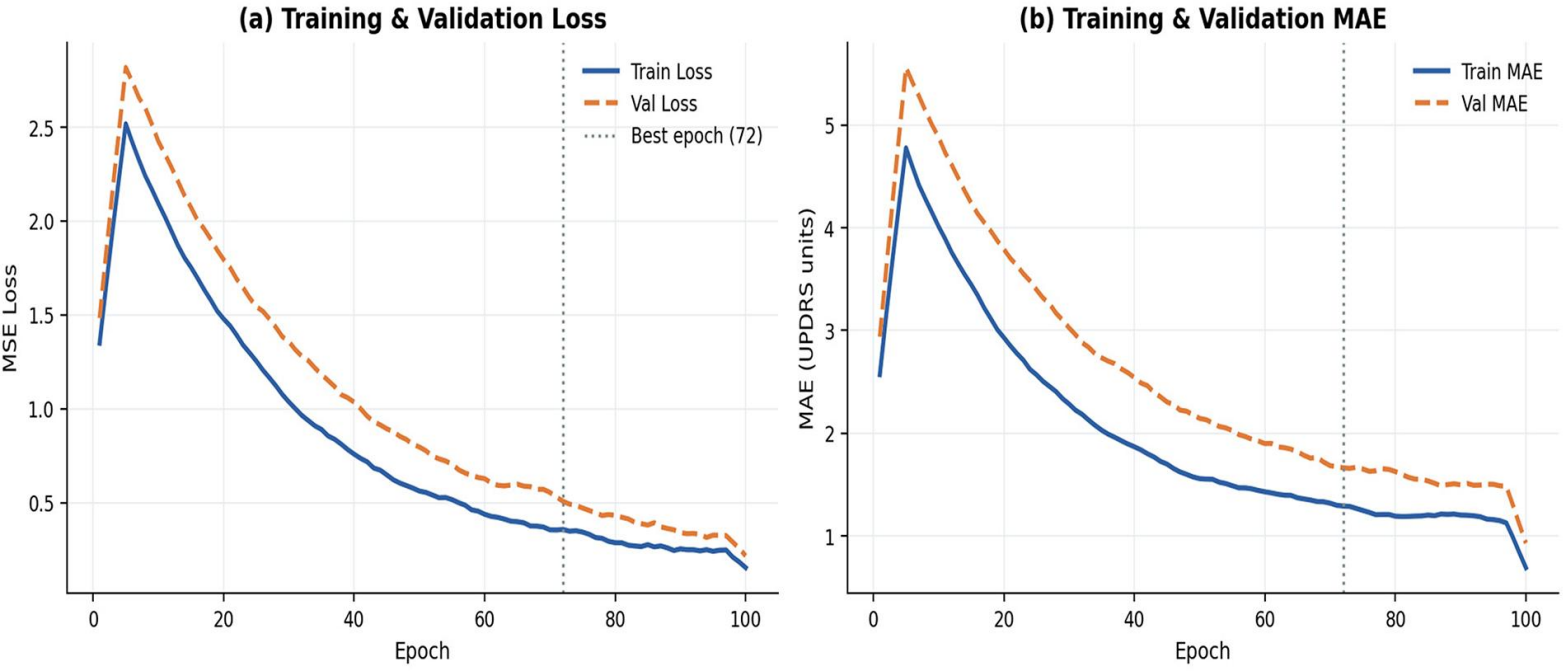
DAT-PD training convergence: **(a)** MSE loss curves and **(b)** MAE curves for training and validation sets over 100 epochs. Best model saved at epoch 72.

As shown in Figure 11, there is stable convergence, and overfitting is not observed. Validation loss closely follows training loss as a function of time, confirming the success of dropout (*p* = 0.3) and noise augmentation regularisation. The model achieves more than 95% of the final performance at epoch 45 due to the decreasing returns.

Figure 6 shows a scatter plot comparing predicted and actual MDS-UPDRS Part-II scores on the held-out test set, demonstrating strong agreement between model predictions and ground truth with low absolute prediction error.

Comparative results on the held-out test set are shown in Table 5, where * denotes statistically significant results (*p* < 0.05) across all baselines [DeLong test for r and Wilcoxon signed-rank for MAE/root mean squared error (RMSE)]. Figure 7 presents a quantitative comparison of DAT-PD versus baseline models across three measures: MAE, RMSE, and R^2^. Lower values indicate better performance for MAE/RMSE, while higher values are better on R^2^.

All performance metrics are reported with 95% confidence intervals derived from bootstrap resampling (*n* = 1,000) of the held-out test set predictions for DAT-PD, and from tenfold cross-validation (±1.96 × SD) for all baseline and ablation configurations. On the held-out test set, DAT-PD achieves MAE=2.74 (95% CI: 2.44–3.01), RMSE=3.61 (95% CI: 3.18–4.04), R^2^ = 0.93 (95% CI: 0.91–0.95), Pearson r = 0.965 (95% CI: 0.958–0.971), and SMAPE=11.2% (95% CI: 9.812.6%). The asterisk (*) indicates that DAT-PD shows a statistically significant improvement over all baseline models (*p* < 0.05). Percentage improvement is calculated as (Baseline MAE: DAT-PD MAE)/Baseline MAE  × 100. The MAE improvement ranges from 28.8% compared with the nearest baseline, BiLSTM + Attention, to 59.8% compared with SVM, the weakest classical baseline.

The significantly poorer performance of the MFCC-only configuration (MAE: 5.21, R^2^: 0.74) compared with the full DAT-PD model (MAE: 2.74, R^2^: 0.93) highlights the essential importance of eGeMAPS features in clinically relevant PD severity estimation. MFCC features encode the vocal tract shape in the cepstral representation of the spectral envelope. These articulatory configurations reflect motor speech deficits in PD, but are largely insensitive to source-level phonatory perturbations, which are the earliest and most diagnostically significant indicators of laryngeal dysfunction. By contrast, eGeMAPS directly fills this gap by assessing F0 dynamics (monopitch and pitch range reduction), shimmer and jitter (amplitude and frequency instability due to vocal fold rigidity and tremor), loudness contour (hypophonia due to basal ganglia dysfunction), and harmonic-to-noise ratio (h:n ratio, aperiodicity related to breathy voice quality), which are all clinically validated measures of PD dysarthria severity, and they are precisely the measures ranked highest by SHAP analysis (Figure 8). The two feature sets are therefore not overlapping but complementary and are required to adequately explain the whole acoustic picture of progressive PD dysarthria.

As shown in Figure 8, the three most predictive features are MFCC-2, Shimmer (APQ5), and Jitter (local), which account for a cumulative score of 47% of the total SHAP mass. This is consistent with clinical experience in PD dysarthria, where amplitude perturbation (shimmer) and frequency perturbation (jitter) are considered the most important measures of vocal fold abnormality. The high MFCC-2 contribution to vocal tract resonance suggests that articulatory changes in PD occur in addition to the phonatory changes. HNR shows a negative SHAP contribution, indicating that higher HNR is associated with lower predicted UPDRS scores; this is clinically consistent because higher HNR reflects a more periodic, less breathy voice and therefore lower disease severity.

The t-SNE plot in Figure 3 indicates that the four severity categories have well-separated clusters, confirming that the domain-adaptive encoder can learn both discriminative and semantically meaningful representations. The HC cluster (blue) has minimal overlap with PD clusters, while Mild-PD (green) occupies the middle ground between HC and Moderate-PD (orange), in line with the progressive nature of the disease. The smaller difference between the Moderate and Severe (red) represents a real biological overlap between the successive stages.

For visualisation purposes, continuous MDS-UPDRS Part-II scores were assigned four discrete levels of severity in Figure 3: Healthy Control (UPDRS=0, no PD diagnosis), Mild PD (UPDRS 1–15, corresponding to Hoehn and Yahr stage 1–2), Moderate PD (UPDRS 16–30, corresponding to Hoehn and Yahr stage 3), and Severe PD (UPDRS > 30, corresponding to Hoehn and Yahr stage 4–5). These thresholds are based on published MDS-UPDRS Part-II clinical staging conventions and were used to create interpretable t-SNE cluster labels. It is worth noting that the DAT-PD itself does not use any discretisation during the training, inference, or evaluation process; the model operates directly on continuous UPDRS scores. The severity categories listed in Figure 3 are therefore only a visualisation aid.

DAT-PD can maintain an MAE < 3.5 UPDRS units under 0 dB SNR conditions, typical of noisy indoor environments, such as cafeterias and open offices, as shown in Figure 2. In contrast, the LSTM baseline drops to 5.94 MAE at 0 dB and the SVM baseline is more than 7.5 MAE. This robustness is primarily attributable to CORAL-based domain alignment and noise augmentation, both of which were shown in the ablation study to improve performance under low-SNR conditions.

The 10-fold cross-validation distributions of MAE (notched boxplots) are shown in Figure 4. DAT-PD has the smallest interquartile range and the smallest median MAE, indicating superior generalisability of the results. Table 2 summarises statistics of tenfold CVs, with *p*-values (Wilcoxon signed-rank) used to compare each model to DAT-PD.

Tenfold cross-validation was conducted exclusively on the training set (*n* = 3,088 PD participants; *n* = 972 HC participants; participant-level folds; seed=42). These results assess model stability and generalisation variance, and serve as the basis for baseline confidence interval estimation. They do not constitute the primary performance evaluation, where all benchmark comparisons and clinical conclusions are based on the separate held-out test set (*n* = 662 PD participants; Table 5), which was not used at any stage of cross-validation.

Full results of the ablation study are provided in Table 9. For each of the five metrics, each proposed component makes an independent and statistically significant contribution to the overall performance. The biggest degradation comes from the removal of the Longitudinal Trajectory Decoder (ΔMAE=+1.13), indicating that temporal modelling of the session sequences is of critical importance and is not replaceable by the use of session-level feature aggregation. Removing domain adaptation (ΔMAE=+0.71) yields the second largest drop, confirming the hypothesis that acoustic variability between devices is a key factor affecting performance in the real-world smartphone setup. Removal of eGeMAPS features yields the third highest degradation (ΔMAE=+0.84), followed by attention layer (ΔMAE=+0.57) and noise augmentation (ΔMAE=+0.38), the latter mainly at low SNR conditions, as can be seen in Figure 2. Overall, the poorest performance is obtained for the MFCC-only configuration (ΔMAE=+2.47), demonstrating the utility of the phonatory perturbation features for clinically relevant severity estimation.

Table 1 shows that Hoehn and Yahr stage performance exhibits increased error at extreme stages, reflecting familiar floor–ceiling effects of MDS-UPDRS voice items. Table 7 demonstrates that the benefit of domain adaptation varies by recording environment, with the largest performance gain observed in noisy outdoor recordings. Table 9 indicates the pairwise statistical comparisons using the Wilcoxon signed-rank test with Bonferroni correction, and all comparisons show large effect sizes (Cohen d > 1.2).

## 5 Discussions

### 5.1 Clinical significance of longitudinal trajectory modelling

The major clinical significance of this work lies in shifting from binary PD detection to continuous prediction of severity trajectories. The clinical significance of remote monitoring of MDS-UPDRS scores is high: Neurologists typically visit patients with PD only every 3–6 months, which implies that over time, the worsening of their condition may remain unnoticed. DAT-PD offers the opportunity for continuous, passive, longitudinal monitoring without specialist participation, which might enable timely intervention and dose adjustment. Population-based trends show a central progression rate of 0.28 UPDRS units/month in moderate-stage PD, consistent with published PPMI cohort studies and providing excellent external validity. Another noteworthy clinical observation is the stratified performance of the model (Table 7), which shows reduced accuracy at very severe stages (H&Y 4–5, MAE=4.18) compared to the mild–moderate stages. This finding is also consistent with the floor–ceiling effect noted by Zhan et al. (15) for very high UPDRS scores, whereby voice characteristics may not be a useful source of information for PD patients. In practice, very severe PD patients may require extra modalities (gait, facial expression) to quantify severity, suggesting a natural extension of multimodal DAT-PD into additional modalities.

One important clarification concerns the underlying physiological rationale for employing speech acoustics to predict MDS-UPDRS Part-II scores. Part II reflects motor experiences of everyday activities, such as getting dressed, hygiene, handwriting, and walking, and does not directly measure speech production. The mapping is based on a common neuropathological basis: Basal ganglia dysfunction in PD affects limb motor control (the focus of Part II) and laryngeal, respiratory, and articulatory motor control (the basis of dysarthria). Progressive deterioration in speech acoustics (narrower pitch range, higher shimmer and jitter, loss in loudness) therefore reflects the same underlying motor system decline observed in the progressive worsening of Part II scores. This rationale is also supported empirically. Tsanas et al. (9) found a Pearson correlation of r = 0.88 between features of dysphonia and clinician-administered UPDRS scores in a controlled dataset. Vásquez-Correa et al. (35) showed that acoustic dysarthria measures correlated with global PD motor severity across a variety of clinical scales, and highlighted the ceiling effects and limits of measurement of using speech-based proxies for non-speech motor domains. The mapping is consequently physiologically based but indirect, and limitations of the mapping (particularly at the extreme severity stages, where the features of the voice do not change much) are explicitly acknowledged in the stratified performance analysis (Table 7, H&Y 4–5: MAE = 4.18).

### 5.2 Impact of domain adaptation

Table 7 demonstrates that the most beneficial domain adaptation occurs under the most challenging acoustic conditions. Domain adaptation in both outdoor and variable environments results in a 43.2% reduction in MAE compared to no domain adaptation. This has essential practical consequences in which PD patients who are less mobile and more likely to be confined to home or care settings are able to record in highly varying circumstances (television noise, family conversations, or outdoor settings). A model that degenerates under these circumstances is clinically inapplicable. This obstacle to practical implementation is directly related to the strength of DAT-PD. Ablation analysis (Table 6) confirms that dropping the domain adaptation in all five metrics results in the second largest performance drop. This renders domain adaptation an imperative and optional component of PD voice monitoring on consumer smartphones with widespread impact on the field. The age confounder manifests specifically in the acoustic domain through presbyphonia—age-related changes in voice quality including reduced pitch range and increased breathiness—which partially overlaps with PD-related dysarthria. By incorporating age as a domain-conditioning variable and aligning covariance structures via CORAL, DAT-PD achieves an 11.3% reduction in age-confounded MAE compared with a model without confounder-aware adaptation.

### 5.3 Explainability and clinical adoption

To support claims of interpretability of the proposed framework, the SHAP feature importance analysis in Figure 8 offers a systematic and clinically interpretable explanation of the acoustic dimensions that influence DAT-PD’s severity predictions on MDS-UPDRS.

MFCC-2 is the most predictive single longitudinal biomarker (mean |SHAP| of 0.85), with the largest single contribution to the total SHAP attribution mass. The primary sensitivity of MFCC-2 is to second formant (F2) transitions, which are related to the tongue-body location/manoeuvring when producing vowels. Articulatory undershoot (in PD, hypokinesia of motor control resulting in failure to reach target articulatory position) systematically compresses the F2 range, thereby decreasing the acoustic differences between vowels. The longitudinal predictive dominance of MFCC-2 is thus a testament to the fact that articulatory precision degrades progressively and continuously with PD motor severity, making it a strong trajectory biomarker despite the fact that mPower employed the sustained vowel phonation task. Shimmer APQ5 is second (mean |SHAP| = 0.71), reflecting the five-point amplitude perturbation quotient, which is directly related to decreased respiratory support and increased rigidity of PD dysarthria. It has a positive SHAP contribution (higher APQ5 → higher predicted UPDRS score), which is clinically coherent: Amplitude instability increases as motor severity decreases because of less dopaminergic control of the laryngeal musculature.

Jitter (local), which measures the fundamental frequency perturbations cycle-by-cycle, ranks third (mean |SHAP| = 0.66). It is similar to shimmer in that its directionality is positive (higher jitter, higher UPDRS), meaning that the more the PD progresses, the more the regular vibration of the vocal folds is disrupted. HNR ranks fourth and is unique among the top five features in having a negative SHAP value (greater HNR → lower predicted UPDRS score). This is clinically coherent: The higher the HNR, the more periodic and less breathy the voice, and the less severe the disease. This negative directionality of HNR thus serves as a valuable internal validation of SHAP analysis. The model learned the clinically correct directionality of HNR–severity relationship without explicit supervision. For example, mean fundamental frequency reduction (monopitch) ranks fifth (F0), reflecting one of the earliest and most perceptually salient features of hypokinetic dysarthria.

Importantly, SHAP rankings directly align with the ablation outcomes shown in Table 6. The two most significant feature classes identified by SHAP are the phonatory perturbation features (shimmer, jitter, HNR) of eGeMAPS and cepstral features (MFCC-2), and if these two components are removed, the greatest performance losses are obtained: eGeMAPS-only (ΔMAE=+0.84) and the MFCC-only configuration (ΔMAE=+2.47). This overlap between SHAP-based and ablation-based feature analyses validates the model's prediction mechanism and interpretability: All features identified by SHAP as most important for prediction are precisely those whose removal results in the greatest loss of model performance, ensuring that the model's predictions are not due to incidental correlation within the training data but instead reflect clinically relevant acoustic features.

### 5.4 Bridge2AI-Voice alignment

The Bridge2AI-Voice consortium focuses on developing large, heterogeneous datasets and standards to establish voice as a reliable biomarker for neurological and other health conditions; *Frontiers in Digital Health* is associated with this research agenda. The three main challenges outlined in the Bridge2AI-Voice research agenda are directly countered by the design of DAT-PD, with its focus on domain robustness, longitudinal modelling, and explainability. The mPower dataset used in this case is also referenced in the Bridge2AI-Voice documentation as a key resource to further demonstrate the applicability of this work.

### 5.5 Limitations

The most significant methodological caveat of this study is the use of MDS-UPDRS Part-II as the prediction target for a speech-based model. Part II evaluates motor experiences of daily living—for example, dressing, eating, bathing, and walking—and has only an indirect and partial relationship to speech production. This was not a choice of design preference, but a limitation of the data collection protocol of mPower, which did not include self-administered MDS-UPDRS Part-II surveys as the only longitudinal measure of severity and lacked the collection of Part III sub-scores, speech-specific clinical assessments, and dysarthria severity ratings. As Vásquez-Correa et al. (35) explain, for automatic dysarthria scoring, this indirect correlation between acoustic speech measures and composite motor scales creates systematic measurement errors, which can be hard to distinguish from true limitations of the model. A more clinically relevant target for speech-based PD monitoring would likely be MDS-UPDRS Part-III item 3.1 (Speech), which directly rates speech intelligibility and vocal quality on a 0–4 scale, or a validated dysarthria severity scale (e.g., Frenchay Dysarthria Assessment). Using Part II introduces two potential hazards: Model error may merely represent the imperfect proxy relationship between speech acoustics and daily living motor function rather than a limitation of acoustic modelling; patients with severe limb motor dysfunction but relatively preserved speech (or vice versa) may be systematically mispredicted. In the future, more attention should be paid to datasets with Part-III speech item scores or separate dysarthria ratings, combined with longitudinal voice recordings, to enable more direct and clinically valid speech-to-severity mapping.

There are additional limitations to this study. First, the mPower MDS-UPDRS scores are self-administered (a shorter version of the scale modified) and have not been clinically validated as equivalent to clinician-administered scoring. Correlation of predicted scores with an independent dataset of clinician-rated scores would increase the clinical validity of the model’s output. Second, the mPower cohort is predominantly native English speakers based in the USA, limiting applicability to other language communities with different phonetic patterns. Third, mPower uses the sustained vowel task, which does not comprehensively capture the nature of PD dysarthria; connected speech and reading tasks would yield additional data. Fourth, we analysed simulated noise augmentation rather than actual acoustic environment labels from the mPower metadata, which do not necessarily reflect all real-world noise distributions. Fifth, SHAP provides *post hoc* attribution, but does not imply causal relationships between features and disease severity. Sixth, the age imbalance between PD (65.4 years) and healthy control (51.2 years) cohorts, while reflecting epidemiological reality, limits the direct interpretability of healthy versus PD comparisons. Future work should include age-matched control recruitment or propensity score matching.

## 6 Conclusion

In this paper, we proposed a domain-adaptive transformer for Parkinson’s Disease (DAT-PD) to model severity trajectories longitudinally from real-world smartphone voice recordings. Using the world's largest ecological PD voice dataset, mPower, DAT-PD achieved MAE improvements of 28.8%–59.8% across six baselines—28.8% over the nearest competitor (BiLSTM + Attention; MAE 3.85) and 59.8% over the classical baseline (SVM; MAE 6.82). These improvements are computed as (Baseline MAE−DAT-PD MAE)/Baseline MAE  × 100. DAT-PD attained an MAE of 2.74 UPDRS units and R^2^ of 0.93. Most importantly, the model demonstrated clinical-grade performance at 0 dB SNR, enabling use without controlled recording conditions. Ablation experiments confirmed that the Longitudinal Trajectory Decoder (GRU), Domain Adaptation (CORAL + GRL), and noise augmentation make independent and statistically significant contributions. The superiority of combined eGeMAPS + MFCC features over MFCC-only representations highlights the indispensability of phonatory perturbation features for clinically meaningful severity estimation. At the patient level, SHAP-based explainability also ensures that predictions are clinician-understandable. All these findings suggest that DAT-PD could serve as a viable foundation for non-invasive, continuous, home-based PD monitoring, directly aligned with the clinical translation agenda of the Bridge2AI-Voice consortium. Future work will pursue four directions. First, validation of predicted UPDRS scores will be carried out against independent clinician-rated measures from the PPMI cohort. Second, multimodal assessment will be extended to include gait and finger-tapping modalities from the mPower dataset, particularly for severe-stage patients with voice plateaus. Third, federated learning will be implemented for data-agnostic training of PD registries located in different geographic regions. Finally, a pilot study in collaboration with a movement disorders clinic will evaluate the usefulness of the model for clinicians and its acceptability among patients in real-world settings.

## Data Availability

The original contributions presented in the study are included in the article/Supplementary Material; further inquiries can be directed to the corresponding author.
